# COVID-19 Lockdown, Sedentarism, Metabolic Alterations, Obesity: Can We Reverse the Domino Effect in Children?

**DOI:** 10.3390/children9060851

**Published:** 2022-06-08

**Authors:** Chiarella Sforza

**Affiliations:** Department of Biomedical Sciences for Health, Università degli Studi di Milano, 20122 Milano, Italy; chiarella.sforza@unimi.it

Some diseases impact more on our personal and social life than other ones depending on various aspects. Among these, mortality rates, transmissibility, kind of patients, understanding of the pathological events, long-term sequels, side effects of the used treatments, are those more commonly considered. From this point of view, the SARS-CoV-2 pandemic greatly influenced the lives of people in all countries, and it is still conditionings activities at almost all ages, including children and adolescents. In this editorial, a selection of the most cited papers dealing with the effects of SARS-CoV-2 infection in children and adolescents, and of its prevention and treatment, will be briefly introduced.

Children were initially considered to be spared by the virus infection or to present only mild symptoms, but later, specific pathologies and syndromes were identified. Among them, the literature reported the “Multi-System Inflammatory Syndrome”, given by a combined set of immune system alterations [1,2]. In addition, while at first neurological disorders were described in adults only, more recent investigations shoved evidence of the neuroinvasive capability of this virus even in newborns, as reviewed by Stafstrom and Jantzie [3].

During the pandemic, children experienced some side effects of lockdown more than adults. In particular, the impressive change to our 24 h scheduled routine was particularly difficult to understand, and therefore to cope with, for children with neurodevelopmental disorders and their families [4] and in general for children who received school-based healthcare services, special services for disabilities, and nutrition programs [5]. The cross-sectional study presented by Nonweiler et al. [4] starts from this altered situation to define new interventions to increase the wellbeing of fragile persons, with a goal for a better quality of life.

One of the additional disorders that the COVID-19 pandemic and related issues such as the lockdown provoked was a dramatic reduction in physical activity [6]. Both adults and children/adolescents had to change their daily routines of activity and rest, as well as their modalities of interaction with peers, teachers, even family members. Notwithstanding the repeated advice given by health authorities and school boards, most young people reduced the duration and intensity of active body movements, increasing the time spent in sedentary occupations [5,6,7,8,9]. The parents were partially responsible for these reductions, as “smart” work often results in longer workdays with a reduced time for the family [7,8]. The lack of open, structured outside facilities also contributed to the widespread rise of indoor inactive occupations [8].

Unfortunately, sedentarism can easily be associated with unhealthy eating behaviors, including increased intake of foods with sugar, salt, and fatty acids and modifications in the timing and modalities of meals, coupled with a reduced caloric expenditure. All these modifications started a domino effect, with a potential huge impact in youth, who experienced metabolic and endocrine changes starting from the modification of the normal sleep/rest routine [6]. An increased number of children and adolescents gained weight and developed metabolic problems, leading them to overweight and obesity [10]. Indeed, a worldwide review including more than 17 million people aged 5 to 25 years found significant changes in body weight in almost all countries [10].

Together with the description of this scenario and its effects on our future health, we should investigate the causes and propose some actions to stop the domino tiles from continuing to fall. For instance, the paper by Bates et al. [6] aptly describes the situation and its origins, but it also goes further, as the pandemic was/is currently under control (at least as we know in the Western countries), and life should return to more healthy habits. A list of possible interventions, starting from the local authorities and involving the individual child/adolescent is reported and discussed [6]; indeed, as sedentary behavior is a bad habit at all ages, a good training program may help to better the physical status of parents as well, possibly reverting the domino tiles’ direction.

Increased physical activity is just one of the suggested tasks, and other interventions might be proposed: a reduction in the time spent with a screen seems to be closely related to an improved health-related quality of life [7]. According to Gilic et al. [9], there is a strong parental influence on physical activity in adolescents, with a positive effect of paternal instruction level and a negative influence of familiar conflicts. As the effect was found before the lockdown and continued during the pandemic, any intervention should be tailored according to a careful assessment of each child/adolescent situation. In contrast, Canadian adolescents aged 14 to 22 years of age demonstrated a correct risk perception of virus infection and appropriately used personal and social preventive measures [11]. They reported a strong desire to protect their families and friends using reliable and up-to-date information.

In conclusion, SARS-CoV-2 infection presents with some differences in adults and children due to both the virus and the preventive mechanisms. As Yang et al. [11] wrote, young citizens who correctly understood the gravity of the disorder may be the first target of selected health interventions, mixing the necessary preventing measures against virus diffusion and useful information about physical activity and healthy eating behaviors. New methods to cope with pandemics are to be defined and tested. The current investigations and reviews can pave the way in targeting today’s adolescents and children: the adults of the nearby future.
